# Survival benefit of adjuvant chemotherapy after resection of Stage I lung adenocarcinoma containing micropapillary components

**DOI:** 10.1002/cam4.7030

**Published:** 2024-02-24

**Authors:** Ying Li, Junfeng Zhao, Ying Zhao, Ruyue Li, Xue Dong, Xiujing Yao, Zhongshuo Xia, Yali Xu, Yintao Li

**Affiliations:** ^1^ Department of Respiratory Oncology Shandong Cancer Hospital and Institute, Shandong First Medical University, and Shandong Academy of Medical Sciences Jinan Shandong China; ^2^ Department of Radiation Oncology Shandong Cancer Hospital and Institute, Shandong First Medical University, and Shandong Academy of Medical Sciences Jinan Shandong China; ^3^ Department of Respiratory Oncology Shandong Cancer Hospital and Institute, Affiliated Hospital of Weifang Medical University, School of Clinical Medicine, Weifang Medical University Weifang Shan Dong China; ^4^ Department of Oncology Zibo Central Hospital, Binzhou Medical university Zibo Shandong China; ^5^ Department of Pathology Shandong Provincial Hospital Affiliated with Shandong First Medical University Jinan Shandong China

**Keywords:** adjuvant chemotherapy, lung adenocarcinoma, micropapillary, non‐small cell lung cancer, radical resection of lung cancer

## Abstract

**Background:**

The usefulness of postoperative adjuvant chemotherapy (ACT) for patients with stage I lung adenocarcinoma with micropapillary (MIP) components remains unclear. We analyzed whether postoperative ACT could reduce recurrence in patients with stage I lung adenocarcinoma with MIP components, thereby improving their overall survival (OS) and disease‐free survival (DFS).

**Methods:**

Data for patients with pathologically confirmed stage I lung adenocarcinoma with MIP components from January 2012 to December 2018 were retrospectively analyzed. OS and DFS were analyzed in groups and subgroups.

**Results:**

Overall, 259 patients were enrolled. Patients who received ACT in stage IA showed significantly better survival than did those with no‐adjuvant chemotherapy (NACT); (5‐year OS 89.4% vs. 73.6%, *p* < 0.001; 5‐year DFS 87.2% vs. 66.0%, *p* = 0.008). A difference was also observed for in‐stage IB patients (5‐year OS 82.0% vs. 51.8%, *p* = 0.001; 5‐year DFS 76.0% vs. 41.11 %, *p* = 0.004). In subgroup analysis based on the proportion of MIP components, patients with 1%–5% MIP components had a significantly better prognosis in the ACT group than in the NACT group (5‐year OS 82.4% vs. 66.0%, *p* = 0.005; 5‐year DFS 76.5% vs. 49.1%, *p* = 0.032). A similar difference was observed for patients with MIP ≥5% (5‐year OS 80.7% vs. 47.8%, *p* = 0.009; 5‐year DFS 73.11% vs. 43.5%, *p* = 0.007).

**Conclusion:**

Among patients with stage I lung adenocarcinoma with MIP components, those who received ACT showed significant survival benefits compared to those without ACT. Patients with lung adenocarcinoma with MIP components could benefit from ACT when the MIP was ≥1%.

## Abstract 2

The need for patients with Stage I lung adenocarcinoma containing micropapillary (MIP) components to receive adjuvant therapy after surgery is unclear. The results of this study suggest that the presence of MIP components is a significant risk factor for poor prognosis. We found that in patients with Stage I lung adenocarcinoma with MIP components, those who received adjuvant chemotherapy showed significant survival benefits.

## 1 INTRODUCTION

Recently, lung cancer incidence and mortality rates have been gradually decreasing due to medical advancements; however, the overall mortality rate is largely attributable to lung cancer.^1^ Adenocarcinoma, the most common histologic type of non‐small cell lung cancer (NSCLC), is responsible for over half of all lung cancers.^2^, ^3^, ^4^ Notably, the most effective and primary treatment for early‐stage lung cancer is still radical surgical resection; however, tumor recurrence and spread after surgical treatment continue to affect the prognosis of patients with early‐stage adenocarcinoma.^5^ Previous study has shown that the prognosis following radical surgical resection varies even among patients with various subtypes of the same disease stage and that the postoperative recurrence of lung adenocarcinoma may be related to its histologic subtype.^6^ In 2011, The International Association for the Study of Lung Cancer, The American Thoracic Society, and the European Respiratory Society proposed a new histologic classification of lung adenocarcinomas that categorized invasive lung adenocarcinomas into five major growth patterns (lepidic, acinar, papillary, micropapillary [MIP], and solid), with a worse prognosis for patients with the MIP subtype and a better prognosis for those with lepidic.^7^, ^8^, ^9^ The MIP component is characterized by tumor cells growing in papillary clusters without a fibrovascular core.^6^, ^7^ After surgical resection, patients with an MIP component are more likely to experience pleural and lymphovascular invasions, as well as lymph node or intrapulmonary metastases.^8^, ^10^, ^11^, ^12^, ^13^ Therefore, the presence of an MIP component in patients with Stage I lung adenocarcinoma can lead to poorer overall survival (OS), disease‐free survival (DFS), and increased risk of recurrence.^6^, ^11^, ^12^ According to prior research, patients with Stage I lung adenocarcinomas should only receive adjuvant chemotherapy (ACT) after radical surgical resection if they have high‐risk factors in stage IB. Patients in stage IA should not receive ACT postoperatively as there is no survival benefit.^14^, ^15^, ^16^, ^17^, ^18^, ^19^ However, regarding whether patients with Stage I lung adenocarcinoma with an MIP component should have ACT added postoperatively, the results of a retrospective analysis by Wang et al. demonstrated that ACT was a favorable prognostic factor for those with Stage IA lung adenocarcinoma with an MIP component and that this group of individuals could benefit from ACT.^6^ Hung et al. retrospective analysis revealed that administering ACT to patients with lung adenocarcinoma with MIP components in Stage IB can reduce the probability of recurrence and, thus, improve patient prognosis.^20^ However, to date, no conclusive evidence exists on whether to add ACT postoperatively in patients with Stage I lung adenocarcinoma with MIP components. Therefore, our study aimed to analyze whether ACT after surgery could reduce recurrence in patients with Stage I lung adenocarcinoma with an MIP component, thereby improving their OS and DFS.

## 2 MATERIALS AND METHODS

### 2.1 Ethics approval

The Ethics Committee of Cancer Hospital Affiliated with Shandong First Medical University gave their approval to this study, eliminating the requirement for informed consent due to the retrospective nature of the research. We declare that patients' information was kept confidential and that we adhered to the principles of the Declaration of Helsinki.

### 2.2 Patient selection

Overall, 259 patients with Stage I lung adenocarcinoma who underwent radical lung cancer resection with pathological results showing MIP from January 2012 to December 2018 at the Affiliated Cancer Hospital of Shandong First Medical University were retrospectively analyzed. The inclusion criteria were as follows: (1) patients with pathologically confirmed lung adenocarcinoma in the presence of MIP and negative surgical margins; (2) those with pathological stage pT1a‐2aN0M0; and (3) those with no obvious cardiopulmonary abnormalities or specific postoperative complications. The exclusion criteria were as follows: (1) postoperative adjuvant therapy other than ACT (e.g., targeted therapy and immunotherapy); (2) pathological Stage II–IV; (3) pathological results of lung adenocarcinoma with a predominantly solid‐type subtype; (4) other high‐risk factors, such as pleural invasion, poorly differentiated tumors, wedge resection, vascular invasion, and unknown lymph node status; and (5) death of non‐cancer causes (Figure S1). The Union for International Cancer Control/American Joint Committee on Cancer 8th edition TNM staging system was used in this study.

### 2.3 Surgical treatment

All patients underwent a multidisciplinary consultation to evaluate the disease and select an appropriate treatment plan before starting treatment, and they were clinically evaluated as resectable NSCLC in the multidisciplinary consultation. The surgical modalities included video‐assisted thoracoscopic surgery or thoracotomy. The methods of lung resection included lobectomy, sleeve resection, segmental resection, and localized resection. All patients were returned to the thoracic surgery department postoperatively, encouraged to cough and expectorate sputum to promote drainage and lung re‐expansion, and instructed to perform early activities.

### 2.4 Adjuvant chemotherapy

All patients were categorized into ACT and no‐adjuvant chemotherapy (NACT) groups based on whether or not they received postoperative ACT. The chemotherapy regimen for patients in the ACT group was intravenous pemetrexed and platinum‐based drugs. Platinum‐based drugs included carboplatin, with an area under the curve value of 5, or cisplatin 25 mg/m^2^ on Days 1–3. Pemetrexed (500 mg/m^2^) was subsequently administered. Patients were treated with chemotherapy every 3 weeks, with an average of four cycles of use.

### 2.5 Follow‐up

All enrolled patients received regular outpatient review and telephone follow‐up after admission, during which they underwent regular physical examination and various diagnostic imaging procedures, including chest‐enhanced computed tomography (CT), positron emission tomography‐CT, ultrasound, endoscopy, magnetic resonance imaging, or whole‐body bone imaging as necessary. For patients whose last case record in the case system was recorded more than 1 month before the cut‐off time of this study, telephone follow‐up was used to complete the collection of patients' clinical data and establish a database for statistical analysis, and they were asked for details of their disease progression and survival. Clinical data collected included whether ACT was performed postoperatively, chemotherapy regimen, age, gender, smoking history, and the presence of spread through air space (STAS). Follow‐up ended on July 31, 2023, with a median follow‐up time of 73 months (range 17–133 months) for all patients.

### 2.6 Study endpoints

The primary endpoint of this study was OS and DFS, with failure mode as a secondary one. OS was defined as the span between radical lung cancer resection and death due to any cause or the last follow‐up. DFS was defined as the time interval from radical lung cancer resection to the first recording to recurrence, death due to any cause, or the last follow‐up. In the failure mode, progression was classified as local regional recurrence (LRR) and distant metastasis (DM), with LRR defined as the recurrence within the lung on the side where the primary tumor was located or local lymph node recurrence. DM was defined as nonregional lymph node metastasis, systemic metastasis, or malignant pleural effusion.^21^

### 2.7 Statistical analysis

Comparisons of continuous variables were made using the independent samples *t*‐test or the rank sum test depending on whether the test sample showed a normal distribution; for categorical variables, the chi‐squared or Fisher's exact test was employed. Kaplan–Meier survival analysis was employed to examine patient DFS and OS, and log‐rank tests were used for comparison. Univariate analyses were performed according to the Cox proportional hazard model, and variables with *p* < 0.05 in the univariate analyses were included in the multivariate analyses to identify independent prognostic factors affecting OS and DFS. All tests were two‐sided, and statistical significance was set at *p* < 0.05. All statistical analyses were performed using R version 4.2.1 software and IBM SPSS Statistics for Windows version 25.0 (IBM Corp., Armonk, NY, USA).

## 3 RESULTS

### 3.1 Patients' baseline characteristics

Overall, 259 patients with lung adenocarcinoma with pathological Stage I underwent radical lung cancer resection at our study institution from January 2015 to December 2018, of which 97 and 162 were in the ACT and NACT groups, respectively. The baseline characteristics are presented in Table 1. Among them, 114 (44.0%) were male, 145 (56.0%) were female, 126 (48.6%) were <60 years old, and 133 (51.4%) were ≥60 years old. For pTNM staging, 153 (59.1%) and 106 (40.9%) patients were in Stages IA and IB, respectively.

**TABLE 1:** Baseline characteristics of enrolled patients.

Variables	Adjuvant therapy		*p*
	ACT (*n* = 97)	NACT (*n* = 162)	
Sex			
Female	48 (49.48%)	97 (59.88%)	0.103
Male	49 (50.52%)	65 (40.12%)	
Age			
<60	51 (52.58%)	75 (46.30%)	0.330
≥60	46 (47.42%)	87 (53.70%)	
History of smoking			
Yes	27 (27.84%)	42 (25.93%)	0.737
No	70 (72.16%)	120 (74.07%)	
History of alcohol			
Yes	22 (22.68%)	33 (20.37%)	0.660
No	75 (77.32%)	129 (79.63%)	
Family history			
Yes	22 (22.68%)	37 (22.84%)	0.976
No	75 (77.32%)	125 (77.16%)	
FEV1%			
≤70	9 (9.28%)	21 (12.96%)	0.370
>70	88 (90.72%)	141 (87.04%)	
T stage			
T1a	19 (19.59%)	45 (27.78%)	0.056
T1b	20 (20.62%)	40 (24.69%)	
T1c	8 (8.25%)	21 (12.96%)	
T2a	50 (51.55%)	56 (34.57%)	
pTNM			
IA	47 (48.45%)	106 (65.43%)	0.007
IB	50 (51.55%)	56 (34.57%)	
Surgical excision method			
Lung lobectomy	89 (91.75%)	140 (86.42%)	0.194
Non‐lobectomy	8 (8.25%)	22 (13.58%)	
Location			
Upper lung	55 (56.70%)	85 (52.47%)	0.695
Middle lung	8 (8.25%)	18 (11.11%)	
Low lung	34 (35.05%)	59 (36.42%)	
Surgical approach			
VATS	93 (95.88%)	156 (96.30%)	0.865
Thoracotomy	4 (4.12%)	6 (3.70%)	
STAS			
Yes	5 (5.15%)	13 (8.02%)	0.379
No	92 (94.85%)	149 (91.98%)	
Predominant subtype			
Lepidic	17 (17.53%)	20 (12.35%)	0.447
Acinar	39 (40.21%)	65 (40.12%)	
Papillary	22 (22.67%)	47 (29.01%)	
Micropapillary	19 (19.59%)	30 (18.52%)	
Amount of intraoperative bleeding^a^	94.43 ± 60.98	94.44 ± 53.92	0.999
Length of surgery^a^	117.86 ± 36.39	115.66 ± 33.74	0.623

Abbreviations: ACT, adjuvant chemotherapy; FEV1%, forced expiratory volume in one second; NACT, no‐adjuvant chemotherapy; STAS, spread through air space; VATS, video‐assisted thoracoscopic surgery.

^a^ Data presented as mean ± standard deviation.

### 3.2 Progression and survival outcomes

**FIGURE 1:**
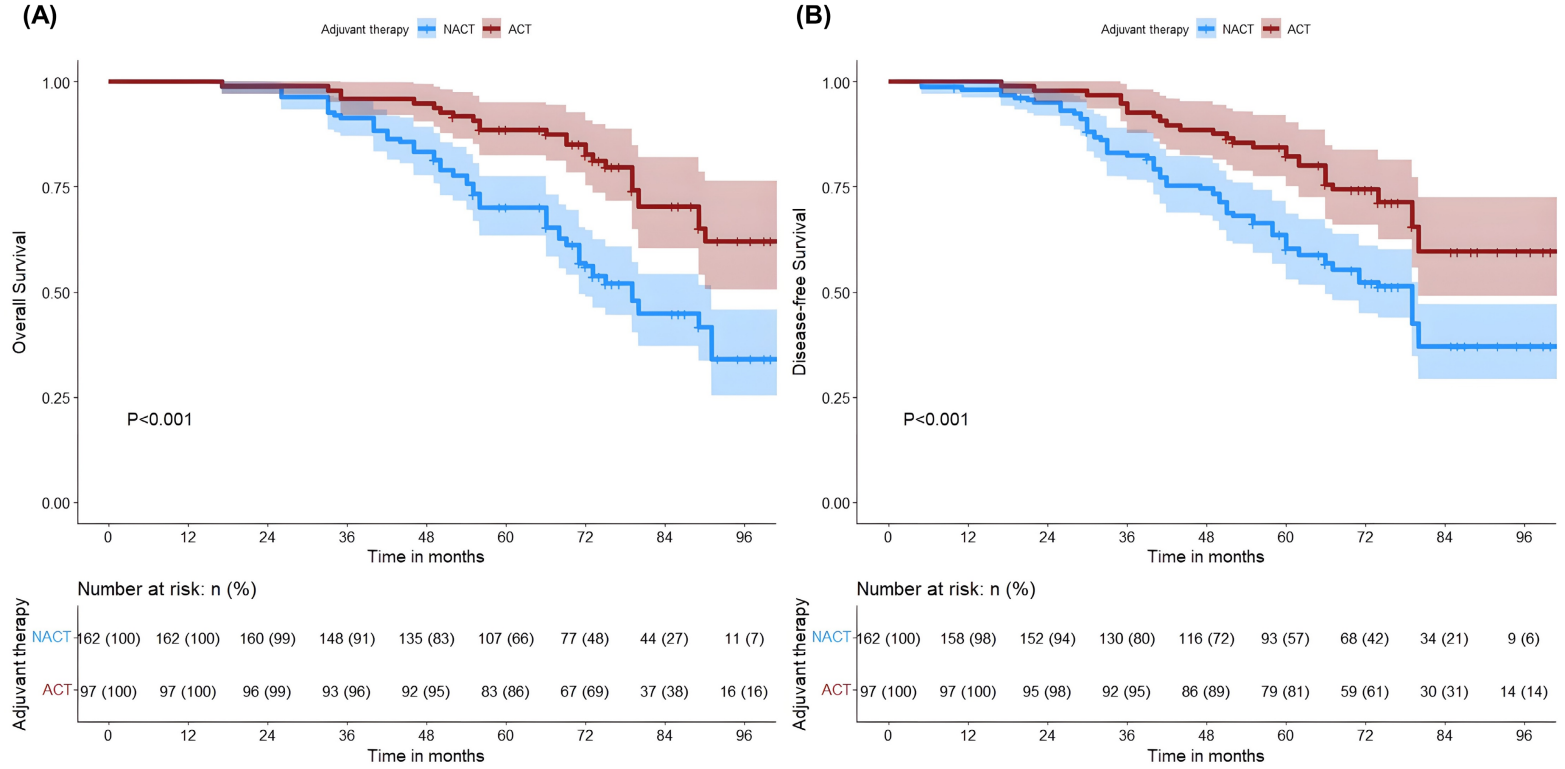
Kaplan–Meier survival analysis of OS (A) and DFS (B) in the ACT and NACT groups. ACT, adjuvant chemotherapy; DFS, disease‐free survival; NACT, no‐adjuvant chemotherapy; OS, overall survival.

**FIGURE 2:**
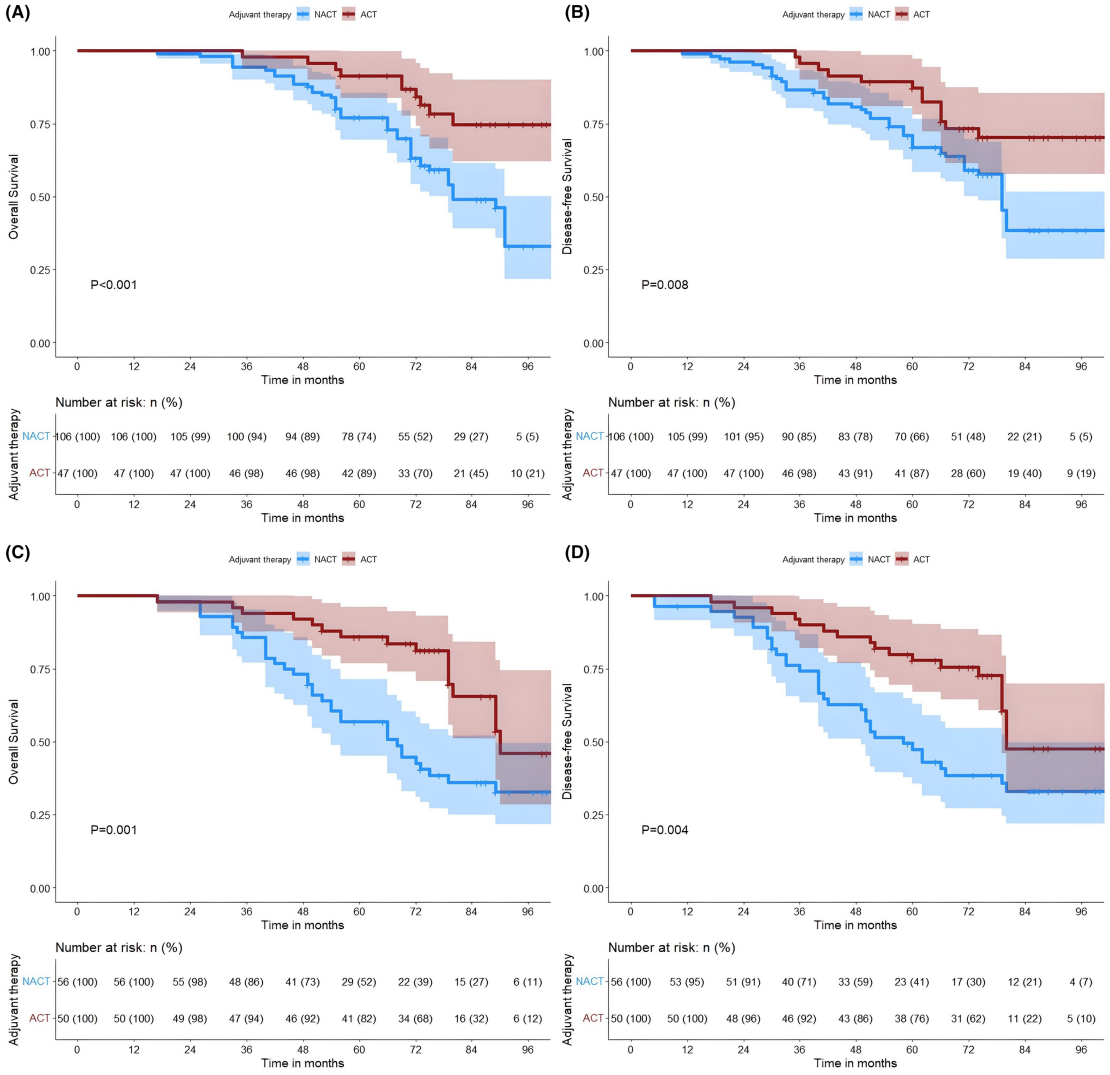
Kaplan–Meier survival analysis of OS (A, C) and DFS (B, D) in the ACT and NACT groups of patients with Stage IA lung adenocarcinoma (A, B) and patients with stage IB lung adenocarcinoma (C, D). ACT, adjuvant chemotherapy; DFS, disease‐free survival; NACT, no‐adjuvant chemotherapy; OS, overall survival.

**FIGURE 3:**
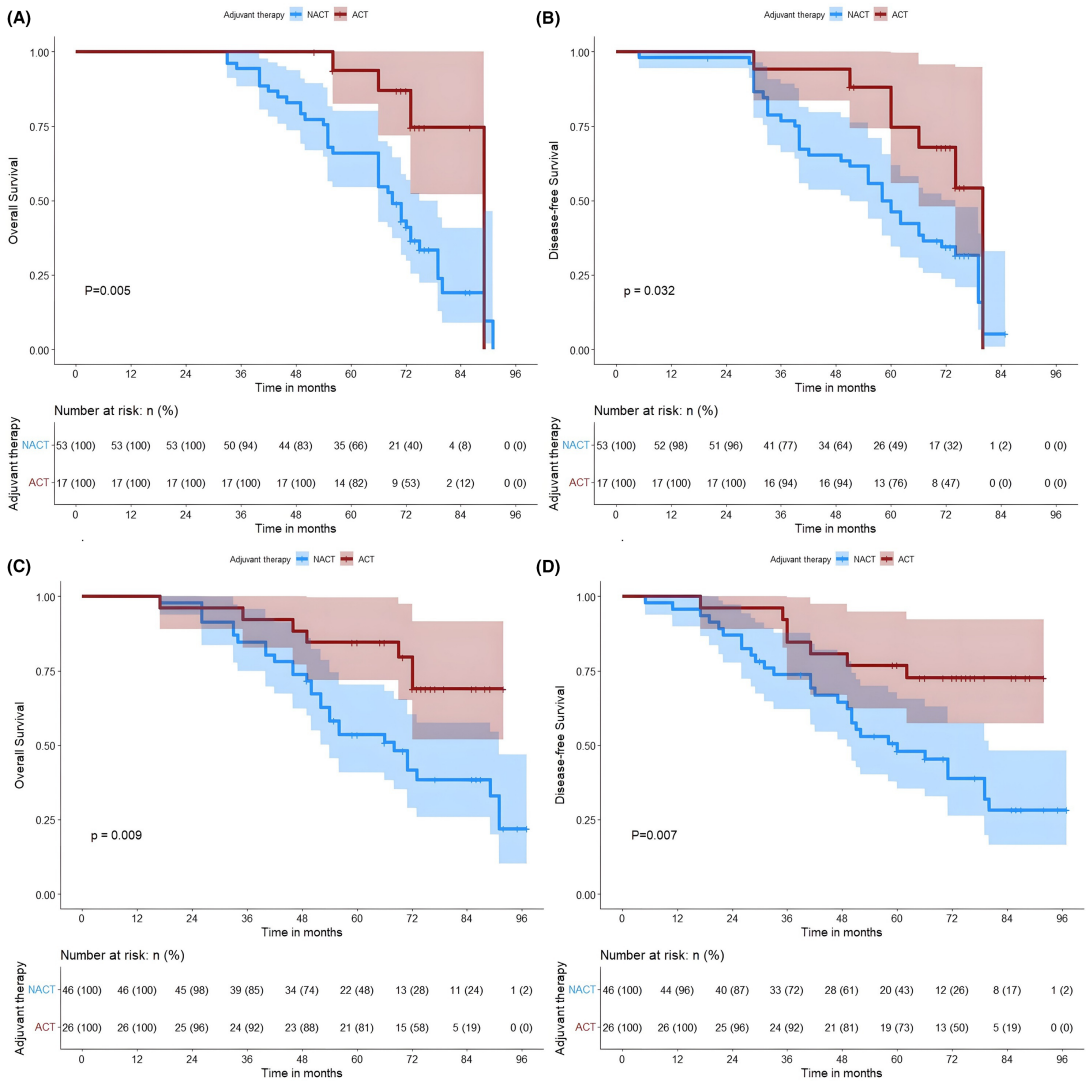
Kaplan–Meier survival analysis of OS (A, C) and DFS (B, D) in the ACT and NACT groups of patients with MIP 1%–5% (A, B); and patients with MIP ≥5% (C, D). ACT, adjuvant chemotherapy; DFS, disease‐free survival; MIP, micropapillary; NACT, no‐adjuvant chemotherapy; OS, overall survival.

**FIGURE 4:**
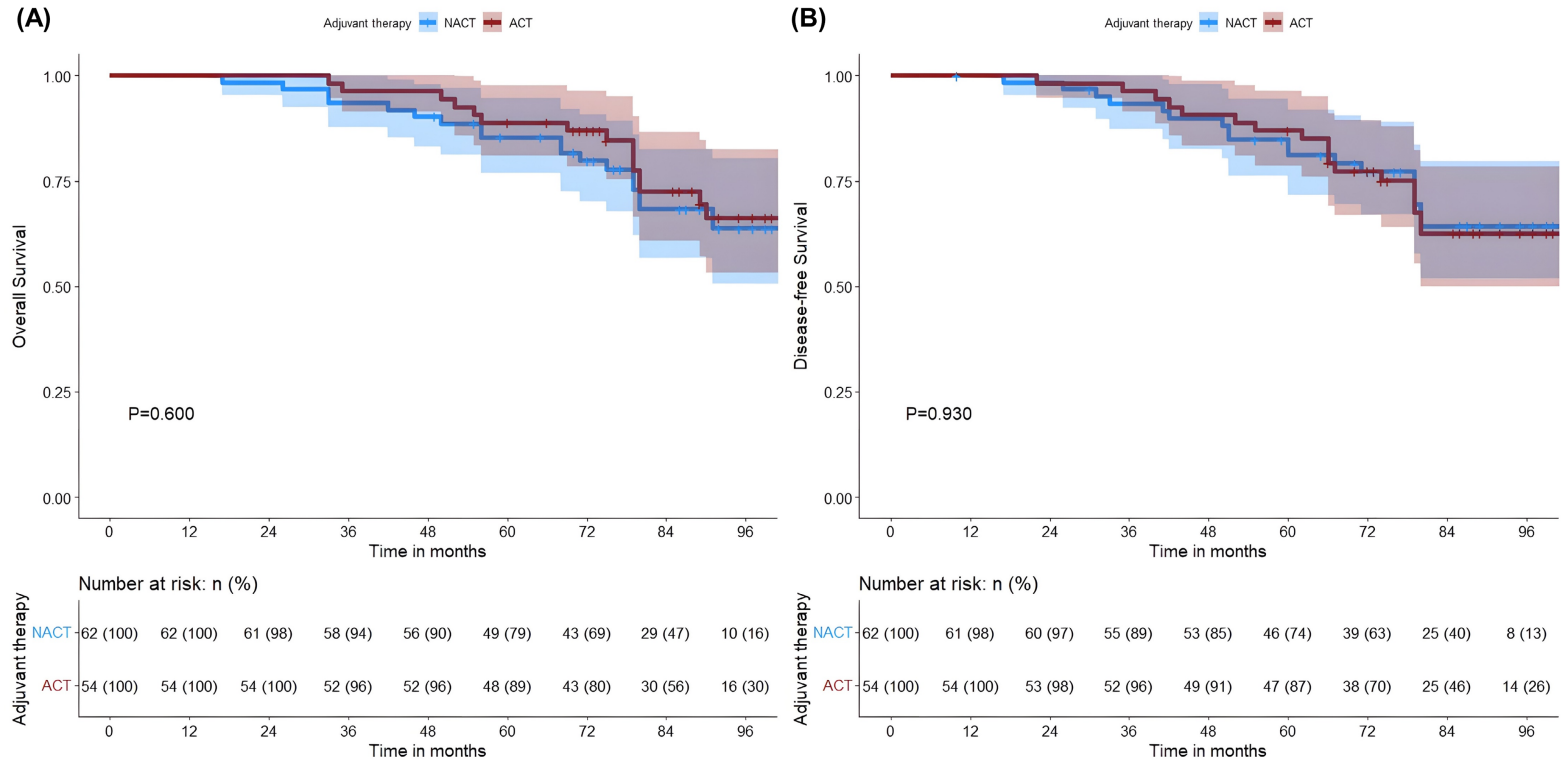
Kaplan–Meier survival analysis of OS (A) and DFS (B) in the ACT and NACT groups of patients with MIP <1%. ACT, adjuvant chemotherapy; DFS, disease‐free survival; MIP, micropapillary; NACT, no‐adjuvant chemotherapy; OS, overall survival.

The median OS of patients in the ACT and NACT groups was not reached and 79 months, respectively. Among patients with Stage I lung adenocarcinoma, we categorized them into Stages IA and IB according to the clinical stage of the disease and analyzed them in separate subgroups. Patients with Stage I lung adenocarcinoma with an MIP component receiving ACT had significant survival advantage (5‐year OS of 85.6% vs. 66.0%, *p* < 0.001; 5‐year DFS of 81.4% vs. 57.4%, *p* < 0.001) (Figure 1). Patients who received ACT in Stage IA had a significantly better OS and DFS than those who underwent NACT (5‐year OS of 89.4% vs. 73.6%, *p* < 0.001; 5‐year DFS was 87.2% vs. 66.0%, *p* = 0.008) (Figures 2A,B); notably, similar differences were observed in patients in Stage IB (5‐year OS was 82.0% vs. 51.8%, *p* = 0.001; 5‐year DFS was 76.0% vs. 41.11%, *p* = 0.004) (Figures 2C,D). Based on the proportion of MIP present, 259 patients with lung adenocarcinoma containing MIP components were categorized into the following three subtypes: (1) MIP <1% (*n* = 117), (2) MIP 1%–5% (*n* = 70), and (3) MIP ≥5% (*n* = 72). In patients who received NACT, we found that OS and DFS were significantly better in those with MIP <1% than in those with MIP ≥1% (*p* < 0.001 and *p* < 0.001, respectively) (Figures S2A,B), whereas no significant difference was observed in those with ACT (*p* = 0.54 and *p* = 0.40, respectively) (Figures S2C,D). In the subgroup analysis, we found that patients with MIP 1%–5% in the ACT group were significantly better than those in the NACT group (5‐year OS was 82.4% vs. 66.0%, *p* = 0.005; 5‐year DFS was 76.5% vs. 49.1%, *p* = 0.032) (Figures 3A,B). Notably, a similar difference was found in patients with MIP ≥5% (5‐year OS 80.7% vs. 47.8%, *p* = 0.009; 5‐year DFS 73.1% vs. 43.5%, *p* = 0.007) (Figures 3C,D). However, we found no benefit of ACT in patients with MIP <1% (5‐year OS 88.9% vs. 79.0%, *p* = 0.600; 5‐year DFS 87.0% vs. 74.1%, *p* = 0.930) (Figure 4).

### 3.3 Failure mode and Cox regression analysis

In the failure mode analysis of the enrolled patients we found that in the subgroup analysis according to the pathological stage, ACT significantly reduced the risk of postoperative DM in patients with Stages I, IA, and IB (*p* = 0.001, *p* = 0.017, and *p* = 0.007, respectively); however, concerning LRR, no significant difference was found (*p* = 0.833, *p* = 0.676, and *p* = 0.593, respectively) (Table S1). In the subgroup analysis with different percentages of MIP, we found that ACT reduced patients' risk of postoperative DM in those with MIP 1%–5% and MIP ≥5% (*p* = 0.043, and *p* = 0.032, respectively); however, we found no benefit of ACT in patients with MIP <1% (*p* = 0.518). Regarding LRR, none of the three subgroups showed a significant advantage with ACT (*p* = 0.061, *p* = 0.912, and *p* = 0.855, respectively) (Table S2). In the Cox analysis, we found that postoperative receipt of ACT (hazards ratio [HR]: 0.485, 95% confidence interval [CI]: 0.312–0.756, *p* = 0.001), MIP <1% (HR: 0.362, 95% CI: 0.238–0.552, *p* < 0.001), and STAS (HR: 13.706, 95% CI: 7.603–24.709, *p* < 0.001) were significantly associated with OS. Similarly, postoperative receipt of ACT (HR: 0.539, 95% CI: 0.359–0.811, *p* = 0.003), MIP <1% (HR: 0.380, 95% CI: 0.255–0.567, *p* < 0.001), and STAS (HR: 11.102, 95% CI: 6.246–19.722, *p* < 0.001) were significantly associated with DFS (Table 2).

**TABLE 2:** Univariate and multivariate analyses of OS and DFS.

Variables	OS univariate analysis		OS multivariate analysis		DFS univariate analysis		DFS multivariate analysis	
	*p*		HR (95% CI)	*p*	*p*		HR (95% CI)	*p*
Age	0.244		—	—	0.109		—	—
History of smoking	0.504		—	—	0.821		—	—
History of alcohol	0.629		—	—	0.794		—	—
FEV1%	0.235		—	—	0.439		—	—
pTNM	0.098		—	—	0.123		—	—
Surgical approach	0.75		—	—	0.793		—	—
Surgical excision method	0.654		—	—	0.704		—	—
T stage	0.050		—	—	0.070		—	—
Adjuvant therapy	<0.001	ACT	0.485 (0.312–0.756)	0.001	<0.001	ACT	0.539 (0.359–0.811)	0.003
		NACT	Ref.			NACT	Ref.	
Proportion of MIP	<0.001	<1%	0.362 (0.238–0.552)	<0.001	<0.001	< 1%	0.380 (0.255–0.567)	< 0.001
		≥1%	Ref.			≥1%	Ref.	
STAS	<0.001	Yes	13.706 (7.603–24.709)	<0.001	<0.001	Yes	11.102 (6.246–19.722)	<0.001
		No	Ref.			No	Ref.	

Abbreviations: 95% CI, 95% confidence interval; ACT, adjuvant chemotherapy; DFS, disease‐free survival; FEV1%, forced expiratory volume in one second; HR, hazard ratio; MIP, micropapillary; NACT, no‐adjuvant chemotherapy; OS, overall survival; STAS, spread through air space.

## 4 DISCUSSION

The highly beneficial and preferred treatment modality for patients with early‐stage lung cancer is surgical resection; however, tumor recurrence is the main reason for surgical treatment failure.^22^ Different pathological subtypes affect the prognosis of patients with Stage I lung adenocarcinoma, and to improve the survival and prognosis of those with early‐stage lung adenocarcinoma, a new classification of lung cancer was proposed by the International Association for the Study of Lung Cancer, the American Thoracic Society, and the European Respiratory Society in 2011.^7^, ^8^, ^9^ Previous studies have demonstrated that patients with predominantly MIP and solid types are associated with poor prognosis and that those with predominantly MIP lung adenocarcinoma have a higher probability of recurrence within 5 years and are frequently associated with metastasis and are prone to STAS, which affects their survival, even when the amount of MIP is low (<1%).^23^, ^24^ These results suggest that we should further improve treatment strategies to enhance the prognosis of patients with Stage I lung adenocarcinoma with MIP. The results of previous studies have shown that ACT significantly improves the survival of patients with NSCLC after radical lung cancer resection.^25^ However, the benefit of undergoing ACT in patients with Stages IA and IB lung adenocarcinoma remains controversial, with some studies suggesting that postoperative ACT is limited to those with Stage II or III NSCLC after resection and that it is ineffective or even leads to worse outcomes in individuals with stage I lung adenocarcinoma, thereby failing to improve the prognosis of those with Stage I lung adenocarcinoma.^17^, ^26^, ^27^ Conversely, whether to routinely use four cycles of ACT postoperatively in patients with Stage I lung adenocarcinoma with MIP components remains debatable, and no study has yet answered definitively whether patients with Stage I lung adenocarcinoma with MIP components can benefit from ACT. Therefore, this study aimed to analyze whether ACT in patients with Stage I lung adenocarcinoma with postoperative pathological findings suggestive of the presence of MIP could improve prognosis, enhance postoperative OS, and reduce tumor recurrence and metastasis to provide a more comprehensive reference for the postoperative clinical treatment of patients with MIP‐containing lung adenocarcinoma in Stages IA and IB.

The ACT group in Stage IA demonstrated a marked superiority over the NACT group in terms of both OS and DFS, and the same was true for Stage IB patients. A subgroup analysis of varying MIP percentages revealed that patients with MIP ≥1% in the ACT group had a significantly better prognosis than those in the NACT group. In the failure mode, in both the subgroups categorized based on pathological stage and MIP content, ACT showed a significant advantage in DM, whereas no significant difference was found in LRR, which suggests that recurrence in patients with lung adenocarcinoma with an MIP component primarily manifests as DM, which is consistent with the results of previous studies.^28^

A study by Tsao et al. found that different histologic subtypes of Stages I–III lung adenocarcinoma had predictive value for ACT benefit and survival, and no advantage of ACT regarding OS was found in patients with Stage I lung adenocarcinomas predominantly of the MIP and solid types.^29^ In this study, we excluded patients who died of non‐cancer deaths, thereby reducing the impact on the results.

Wang et al. found that those with MIP‐predominant lung adenocarcinoma had poorer survival and that ACT significantly improved the prognosis of those with MIP‐predominant adenocarcinomas in Stage IA (no vs. chemotherapy: HR: 2.054, 95% CI: 1.085–3.886, *p* = 0.027).^6^ Our study's results are in agreement with this. Our results show that ACT significantly improves the prognosis of patients with Stage IA adenocarcinoma with MIP; however, this is different from the results of the LACE pooled analysis, which demonstrated that the postoperative administration of cisplatin‐containing chemotherapy was detrimental to the prognosis of patients with Stage IA NSCLC after radical resection.^30^ The study's limited sample size of patients with Stage IA lung adenocarcinoma could be the cause of this result. The advantages of ACT are evident in our results, and we consider that it may be because of the predominance of patients with MIP ≥5% in Stage IA adenocarcinoma, which has a poorer prognosis; thus, ACT shows an advantage.

The findings of our study revealed that ACT significantly increased the OS and DFS of patients with Stage IB adenocarcinoma. This is consistent with the results of Huang et al. that ACT improves OS and decreases the probability of recurrence in patients after pneumonectomy in Stage IB adenocarcinoma. The difference is that they chose the endpoint of no probability of recurrence; however, it can be concluded that ACT is effective for patients with Stage IB adenocarcinoma with MIP components.^20^ Similarly, Strauss GM et al.'s study showed that ACT could be beneficial in those with Stage IB adenocarcinoma, yet it cannot be employed as a regular standard of treatment postoperatively for those with Stage IB NSCLC.^15^ It appears that adjuvant therapy is a viable option for those with Stage IB tumors ≥4.0 cm, as indicated by their findings. In contrast to our study, where we focused on the effect of the MIP component on the effectiveness of ACT in patients with Stage IB adenocarcinoma, they preferred the effect of tumor size on ACT, and such results are also worth considering.

Additionally, our study was grouped according to the MIP component. Survival analysis showed that among patients with NACT, the prognosis of those with MIP <1% was significantly better than that of those with MIP ≥1%; however, no difference was found among patients with MIP 1%–5% and MIP ≥5%, which we considered might be due to the study population being limited to the earliest stage of lung adenocarcinoma, namely, Stage I. The results showed that the prognosis of patients with MIP <1% was significantly better than that of those with MIP ≥1%. Because the MIP component is highly malignant and grows rapidly, Stage I lung adenocarcinoma may become a major component during development, even if MIP contains only 1%–5%. Moreover, we excluded patients with lymph node metastasis. The MIP component increases the risk of lymph node metastasis; the larger the MIP component, the more probable it is that lymph node metastasis will occur.^30^ The exclusion of patients with lymph node metastasis may have excluded some patients with poor prognoses. No difference in prognosis was observed in patients with ACT, demonstrating that ACT significantly improved the prognosis of those with MIP components. Our results suggest that ACT does not significantly improve the prognosis of patients with MIP <1%. This is consistent with previous studies, which defined MIP as positive when it accounted for >1% of the whole tumor and MIP <1% as negative, indicating patients with no MIP component.^30^ This is because, as found by Lee et al., patients with lung adenocarcinoma with MIP <1% had significantly better OS than those with MIP 1%–5% and MIP ≥5%.^31^ Therefore, whether or not to receive ACT may not be significantly different in patients with MIP <1%. However, some studies have suggested that even in the case of low MIP (<1%), the survival of patients is affected.^23^ Therefore, further research is required to decide whether ACT should be administered postoperatively to this group of patients with low MIP to select an appropriate treatment plan for them. In our study, ACT significantly improved the survival and prognosis of patients in the 1%–5% and ≥5% MIP segments. Previous studies have demonstrated that even a small percentage of MIP (≥1%) remains an important risk factor for poor OS and DFS.^32^, ^33^, ^34^ Therefore, ACT in this group of patients can improve their prognosis and reduce the probability of postoperative metastatic recurrence.

Two factors may be associated with the susceptibility of those with Stage I MIP to metastasis following surgical resection. First, the tendency to develop STAS. Normally, apoptosis (anoikis) occurs when epithelial cells detach from the extracellular matrix. The MIP component's cells, in comparison, likely developed anoikis resistance (survival after detachment) and promoted anchorage‐independent growth (ability to proliferate in the vascular system and lymphatic circulation).^28^ Histologically, MIP‐like clusters separate from the main tumor and may float in the air. For example, Kamiya et al., based on pathological serial sections, determined that the clusters in the complex structure of MIP appeared to extend into the air and that the lungs contained sufficient air that the cells comprising the MIP clusters may easily and extensively extend beyond the main tumor, potentially leading to the occurrence of STAS.^35^ Watanabe et al. recommended a thorough pathological examination to clarify whether the MIP component floats close to the stump in the case of partial resection because lung adenocarcinomas with MIP components can spread farther than is usually expected through airborne dissemination.^36^ However, recent research on the cause of STAS also suggests that it is due to squeezing by the surgeon during intraoperative exploration or postoperative examination of the specimen. Therefore, to locate small tumors within the lobes of the lungs, the surgeon will press on the lung tissue with his hand to feel for resistance. This undoubtedly places mechanical stress on the tumor, which may be somewhat adherent, making it easy for clusters of cells to spread from the periphery of the tumor into the adjacent airspace.^37^ Therefore, further research on the mechanisms of STAS is needed to inform clinicians' medical decisions. Another important aspect to consider is the presence of micrometastases. Currently, commonly used clinical tests cannot detect micrometastases that have spread to distant parts of the body, which affects the assessment of the true stage of tumor development and leads to the occurrence of late‐stage metastases, causing poor outcomes. Lymph node metastasis of the tumor is an important factor in determining the treatment of NSCLC, and studies have found a strong correlation between MIP and lymph node metastasis.^30^, ^38^ For example, Kamiya et al. demonstrated that the presence of MIP components may be associated with a high frequency of micrometastases.^35^ It has also been stated that MIP is one of the important factors associated with a high risk of early lymph node micrometastasis.^39^, ^40^ Lymph node micrometastases are a type of occult lymph node metastasis (used to describe metastases that cannot be diagnosed using standard clinical and pathological methods in negative lymph nodes), which are isolated tumor cells or clusters of cells with a maximum size of ≤0.2 mm found within a lymph node.^41^ In patients with lymph node micrometastases, occult lymph node metastases cannot be detected preoperatively using CT scanning or 18F‐fluorodeoxyglucose positron emission tomography scanning and frequently demonstrate a high false‐negative rate.^42^ During histological evaluation, small lesions of metastatic tumor cells are also difficult to identify on routine hematoxylin and eosin slides. Increasing evidence shows that lymph node micrometastases present a poor prognosis for patients with malignant tumors.^43^, ^44^ Several methods are available for detecting lymph node micrometastases, including immunohistochemistry and reverse transcriptase polymerase chain reaction.^45^, ^46^ Moreover, most studies have demonstrated that patients with lymph node micrometastases detected using immunohistochemistry are significantly associated with poorer survival and that immunohistochemistry is a standard, reliable method for detecting lymph node micrometastases in lung cancer.^47^

This study had some limitations. First, because of the retrospective nature of the study, there was an inevitable bias regarding the administration of ACT and the treatment regimen. Second, the sample size was not large enough for an effective evaluation of the survival benefits of ACT. Therefore, prospective and randomized clinical trials are necessary to further validate the benefit of ACT in patients with lung adenocarcinoma with MIP components, which is a high‐risk subtype. In conclusion, compared with patients with Stage I lung adenocarcinoma with MIP components in the NACT group, those in the ACT group had significant benefits, with markedly improved DFS and OS. Patients with lung adenocarcinoma with MIP could benefit from ACT when the MIP components were ≥1%.

## AUTHOR CONTRIBUTIONS

**Ying Li:** Data curation (equal); formal analysis (equal); investigation (equal); methodology (equal); validation (equal); writing – original draft (equal). **Junfeng Zhao:** Data curation (equal); investigation (equal); methodology (equal); software (equal); visualization (equal). **Ying Zhao:** Data curation (equal); resources (equal). **Ruyue Li:** Formal analysis (equal); visualization (equal). **Xue Dong:** Data curation (equal); validation (equal). **Xiujing Yao:** Formal analysis (equal); software (equal). **Zhongshuo Xia:** Data curation (equal); resources (equal). **Yali Xu:** Project administration (equal); writing – review and editing (equal). **Yintao Li:** Funding acquisition (lead); investigation (lead); methodology (lead); project administration (lead); supervision (lead); writing – review and editing (lead).

## FUNDING INFORMATION

This work was supported by the National Natural Science Foundation of China [grant number 82373044] and the Natural Science Foundation of Shandong Province [grant number ZR2022LSW001, ZR2023LSW023].

## CONFLICT OF INTEREST STATEMENT

The authors declare that they have no competing interests.

## ETHICS STATEMENT

This study was approved by the Ethics Committee of Cancer Hospital Affiliated with Shandong First Medical University, which waived the need for informed consent because of the retrospective nature of the study. We declare that patients' information will be kept confidential and that we adhered to the principles of the Declaration of Helsinki.

## Supporting information


Figure S1.

Figure S2.

Table S1.

Table S2.

## Data Availability

The datasets used and analyzed in this study are available from the corresponding author upon request.
