# Supplementary figures and images for: Survival benefit of adjuvant chemotherapy after resection of Stage I lung adenocarcinoma containing micropapillary components
Source: Cancer Med. 2024 Feb 24;13(3):e7030. doi: 10.1002/cam4.7030 (PMC10891450; doi:10.1002/cam4.7030)

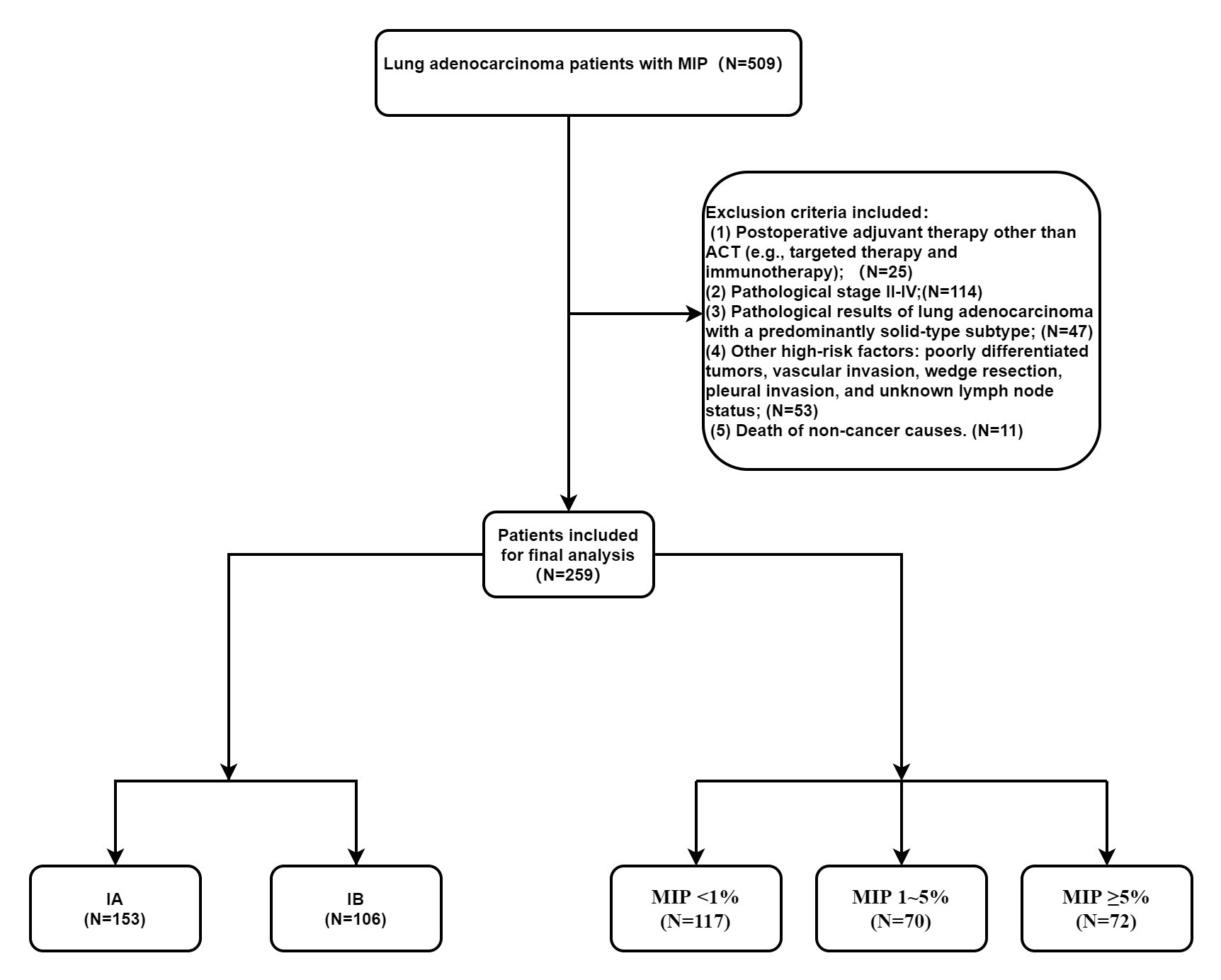

Supplement: Supplementary file 1 — Figure S1. [file CAM4-13-e7030-s002.jpg]

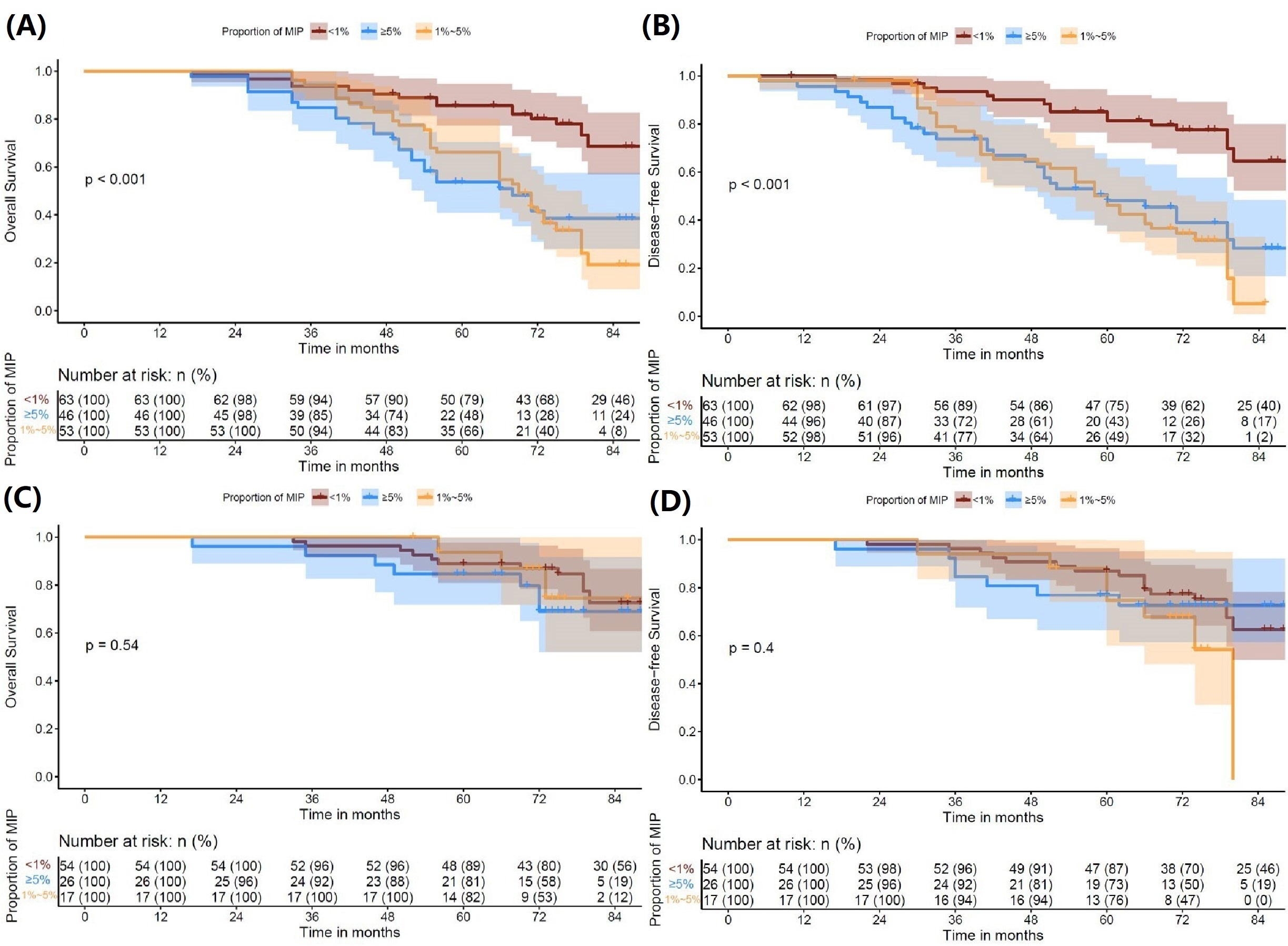

Supplement: Supplementary file 2 — Figure S2. [file CAM4-13-e7030-s004.jpg]
